# Cytoplasmic Tail Truncation Stabilizes S1-S2 Association and Enhances S Protein Incorporation into SARS-CoV-2 Pseudovirions

**DOI:** 10.1128/jvi.01650-22

**Published:** 2023-02-15

**Authors:** Lizhou Zhang, Nancy Hom, Amrita Ojha, Klaus N. Lovendahl, Huihui Mou, Kelly K. Lee, Hyeryun Choe

**Affiliations:** a Department of Immunology and Microbiology, Scripps Biomedical Research at the University of Florida, Jupiter, Florida, USA; b Department of Medicinal Chemistry, University of Washington, Seattle, Washington, USA; Loyola University Chicago

**Keywords:** cytoplasmic tail, entry, infectivity, pseudovirus, S protein, S1 shedding, SARS-CoV-2, spike density

## Abstract

Truncations of the cytoplasmic tail (CT) of entry proteins of enveloped viruses dramatically increase the infectivity of pseudoviruses (PVs) bearing these proteins. Several mechanisms have been proposed to explain this enhanced entry, including an increase in cell surface expression. However, alternative explanations have also been forwarded, and the underlying mechanisms for the severe acute respiratory syndrome coronavirus 2 (SARS-CoV-2) S protein remain undetermined. Here, we show that the partial or complete deletion of the CT (residues 19 to 35) does not modify SARS-CoV-2 S protein expression on the cell surface when the S2 subunit is measured, whereas it is significantly increased when the S1 subunit is measured. We also show that the higher level of S1 in these CT-truncated S proteins reflects the decreased dissociation of the S1 subunit from the S2 subunit. In addition, we demonstrate that CT truncation further promotes S protein incorporation into PV particles, as indicated by biochemical analyses and cryo-electron microscopy. Thus, our data show that two distinct mechanisms contribute to the markedly increased infectivity of PVs carrying CT-truncated SARS-CoV-2 S proteins and help clarify the interpretation of the results of studies employing such PVs.

**IMPORTANCE** Various forms of PVs have been used as tools to evaluate vaccine efficacy and study virus entry steps. When PV infectivity is inherently low, such as that of SARS-CoV-2, a CT-truncated version of the viral entry glycoprotein is widely used to enhance PV infectivity, but the mechanism underlying this enhanced PV infectivity has been unclear. Here, our study identified two mechanisms by which the CT truncation of the SARS-CoV-2 S protein dramatically increases PV infectivity: a reduction of S1 shedding and an increase in S protein incorporation into PV particles. An understanding of these mechanisms can clarify the mechanistic bases for the differences observed among various assays employing such PVs.

## INTRODUCTION

Severe acute respiratory syndrome coronavirus 2 (SARS-CoV-2) encodes 15 nonstructural proteins as well as 4 structural proteins. Of these, only the structural proteins, spike (S), membrane (M), envelope (E), and nucleocapsid (N), are incorporated into the virion. The N protein is essential for the encapsidation of the 30 kb positive-sense RNA genome, the M and E proteins contribute to virus assembly and budding via interactions with other viral proteins (1, 2), and the S protein mediates entry into the target cell.

For successful entry, the S protein needs to be activated by two sequential proteolytic cleavages. The first cleavage divides the S protein into the S1 and S2 subunits, of which S1 binds the receptor angiotensin-converting enzyme 2 (ACE2) and S2 mediates membrane fusion (3). In the case of the first SARS-CoV that emerged 2 decades ago, cleavage at the S1-S2 junction is accomplished in target cells by a cell surface protease, TMPRSS2, or lysosomal proteases, cathepsins (4–8). In contrast, for SARS-CoV-2, cleavage is carried out by furin, a Golgi-resident protease, in infected cells during virus maturation. The second cleavage occurs at a site internal to the S2 subunit, termed the S2′ site, and is carried out by TMPRSS2 or cathepsins for both SARS-CoV and SARS-CoV-2 when they reach the target cells. This second cleavage releases the fusion peptide that is required for subsequent fusion between the cellular and viral membranes (3).

Like SARS-CoV-2, the entry glycoproteins of many viruses are cleaved into the surface and transmembrane subunits prior to virus release from infected cells. For most of these viruses, the two subunits remain associated until the receptor-binding domain (RBD) located in the surface subunit binds the receptor. Receptor binding induces conformational changes in the entry glycoprotein and leads to the dissociation of the surface subunit and subsequent membrane fusion mediated by the transmembrane subunit. In the case of the original Wuhan-Hu-1 strain of SARS-CoV-2, the S1-S2 association is weak, and thus, S1 was easily shed from the spikes (9). To overcome this problem, the virus acquired the D614G mutation early in the pandemic, which stabilized the S1-S2 association and increased virus infectivity (9–12).

Another modification of the SARS-CoV-2 S protein that increases virus infectivity is cytoplasmic tail (CT) truncation. The understanding that CT truncation of viral entry glycoproteins enhances pseudovirus (PV) infectivity originated decades ago from intriguing observations that lentiviruses grown in human T cell lines acquired a premature stop codon in their CTs (13–16). Multiple subsequent studies reported that CT truncations of the entry glycoproteins of various viruses enhanced PV infectivity (17–21). We also showed that a 19-amino-acid truncation of the SARS-CoV S protein enhanced PV infectivity (21). Mechanistic studies showed that CT truncation upregulated the cell surface expression of the entry glycoproteins (20, 22). These observations have been interpreted to suggest that the removal of the endoplasmic reticulum (ER) retention signal present at the carboxy-terminal end of the CT was responsible for the elevated expression on the cell surface. However, other studies did not observe increased expression of the CT-truncated glycoproteins on the cell surface (23, 24). In addition, even if an increase was observed, the degree of the increase was modest, and thus, it is difficult to explain the dramatic changes in PV infectivity. An alternative mechanism, a conformational change in the ectodomain, was also proposed for various enveloped viruses to explain the increased fusogenicity upon CT truncation of their entry glycoproteins (20, 23, 25–27).

Here, we show that CT truncation of the SARS-CoV-2 S protein modestly increases cell surface expression when both S1 and S2 are measured, but no increase is observed when only S2 is detected. When only S1 is detected, however, CT-truncated S protein expression on the cell surface is substantially elevated. Further investigation shows that these differences are contributed by reduced S1 shedding in the CT-truncated S protein. We also demonstrate through biochemical and cryo-electron microscopy (cryo-EM) studies that PVs bearing the CT-truncated S protein exhibit much higher spike densities. Together, our studies show that CT truncation of the S protein enhances PV infectivity by decreasing S1 dissociation and increasing S protein incorporation into PV particles.

## RESULTS

### Cytoplasmic tail truncation of the S protein does not change SARS-CoV-2 PV production but dramatically enhances PV infectivity.

**FIG 1:**
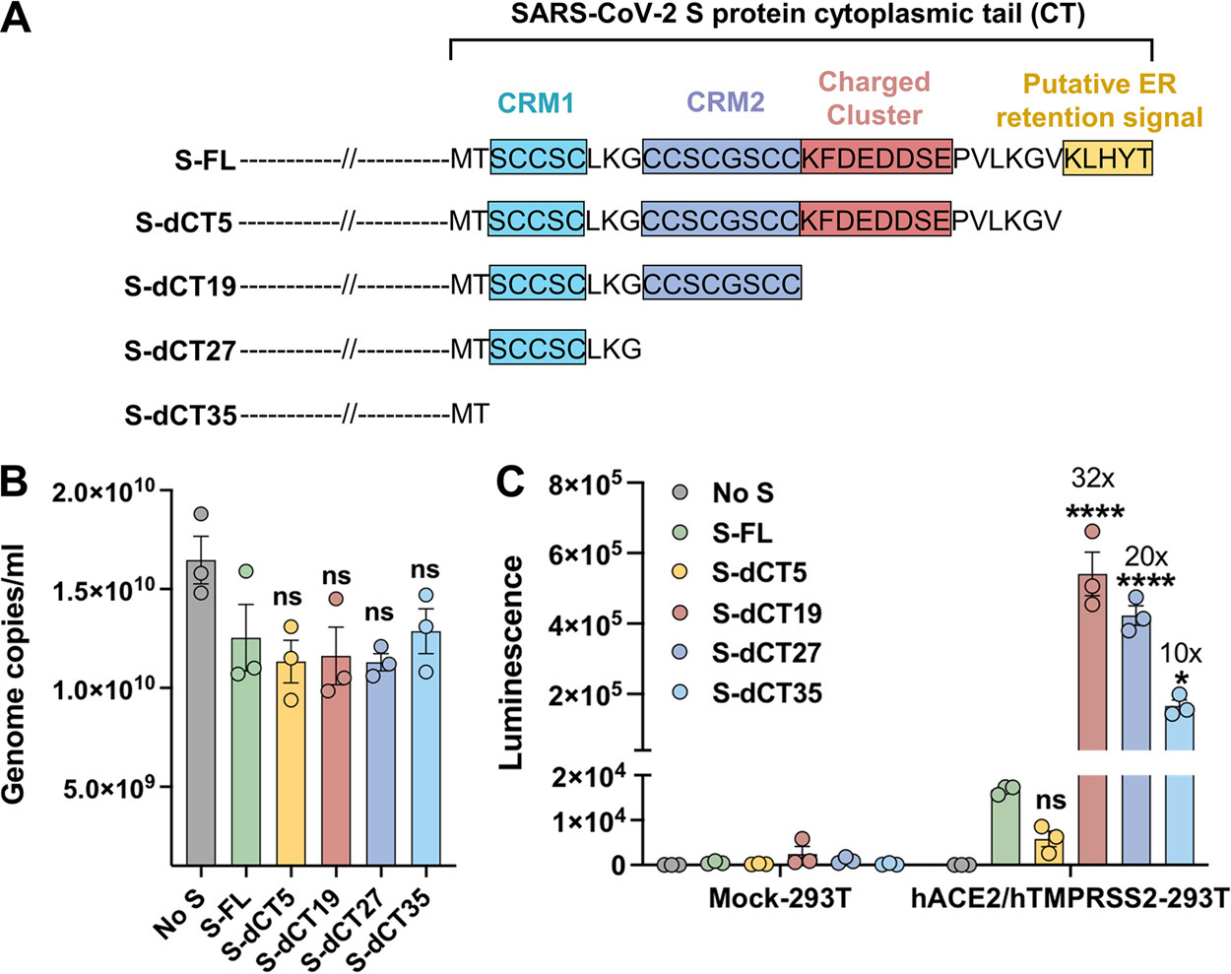
Cytoplasmic tail truncation of the S protein does not change SARS-CoV-2 PV production but dramatically enhances PV infectivity. (A) Diagram representing the CT truncation variants of the S protein used in this study. (B) MLV PVs bearing the full-length S protein (S-FL) or its CT truncation variants (S-dCTs) and expressing firefly luciferase were produced from HEK293T cell transfection, and their titers were quantified by RT-qPCR. (C) Infectivity of the same PVs in parental HEK293T cells or the same cells expressing human ACE2 and TMPRSS2. Cells were infected for 1 h with 5 × 10^8^ genome copies per well in a 48-well plate, and infection levels were assessed by measuring luminescence at 24 h postinfection. Panels B and C show mean values ± SEM from three independent experiments conducted with three independently prepared PVs, and statistical significance was calculated by two-way ANOVA using Sidak’s multiple-comparison test (*, *P* < 0.05; ****, *P* < 0.0001; ns, not significant). CRM, cysteine-rich motif.

The CT of the SARS-CoV-2 S protein consists of 37 amino acids that contain four motifs, as shown in Fig. 1A: a putative ER retention signal (KXHXX) (28, 29), a charged cluster (KFDEDDSE) (30), and two cysteine-rich motifs (CRMs), CCSCGSCC and SCCSC (30–32). To better understand the role of the CT in SARS-CoV-2 infectivity, we made truncation variants of the S protein (S-dCTs) in which these motifs were sequentially deleted, and we compared them to the full-length S protein (S-FL). PVs expressing firefly luciferase (FLuc) and bearing these S-dCTs (PV-dCTs) were produced from HEK293T cell transfection. PVs bearing S-FL (PV-FL) or no S protein were used as controls. To assess the effect of CT truncation on the PV yield, PV titers were quantified by reverse transcription-quantitative PCR (RT-qPCR). Figure 1B shows that these PVs were produced at comparable levels, indicating that CT truncation did not affect PV production.

Next, we assessed their entry efficiency by infecting HEK293T cells stably expressing human ACE2 and TMPRSS2 (hACE2/hTMPRSS2-293T cells) with the same genome copy numbers. HEK293T cells transduced with empty vectors but selected with drugs in the same way as for hACE2/hTMPRSS2-293T cells were used as negative controls for infection (Mock-293T cells). As shown in Fig. 1C, S-dCT5 unexpectedly decreased PV entry compared to S-FL, indicating that the KLHYT motif, a putative ER retention signal, may play its role only in the context of other motifs. In contrast, S-dCT19 increased PV entry by more than 30-fold, which is consistent with previous reports that a deletion of the last 13 to 21 amino acids from the carboxy terminus of the CT enhances PV infectivity (23, 24, 33, 34). S-dCT27 and S-dCT35, which lack one or both CRMs, respectively, also increased PV entry compared to S-FL but to a lesser degree than did S-dCT19, which contains both CRMs (Fig. 1C). Together, these data confirm that CT truncations, except dCT5, substantially enhance the PV entry efficiency and suggest that although the CRMs may play a role in PV infectivity, they are not essential for either PV production or entry.

### Cytoplasmic tail truncation does not increase S2 levels but significantly increases S1 levels on the cell surface.

**FIG 2:**
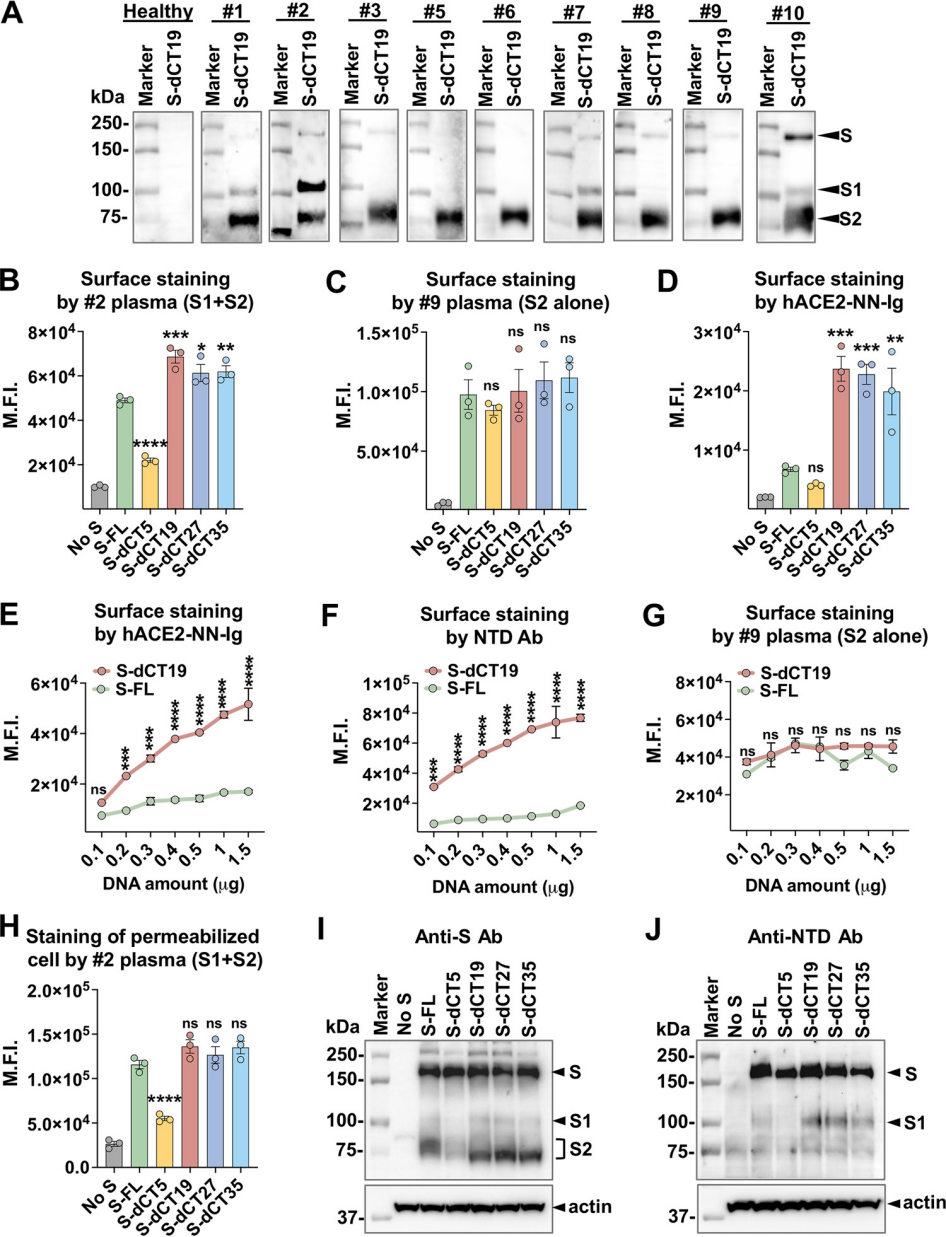
Cytoplasmic tail truncation does not increase S2 levels but significantly increases S1 levels on the cell surface. (A) Ten convalescent-phase plasma samples derived from individuals infected with SARS-CoV-2 early in the pandemic were screened by Western blot analyses for their ability to recognize the S1 and S2 bands of sucrose-pelleted PV-dCT19. (B to D) HEK293T cells grown on 6-well plates were transfected with 0.3 μg of a plasmid encoding the indicated S-FL or S-dCT protein, and their cell surface expression levels were assessed at 42 h posttransfection using plasma sample 2 (at a 1:200 dilution), which recognizes both S1 and S2 (B); plasma sample 9 (at a 1:200 dilution), which recognizes only S2 (C); or 3 μg/mL of hACE2-NN-Ig, which binds only S1 (D). Shown are the mean fluorescence intensity (M.F.I.) values ± SEM from three independent experiments. (E to G) HEK293T cells on 6-well plates were transfected with the indicated amounts of the plasmid encoding S-FL or S-dCT19, and cell surface staining was conducted with 3 μg/mL hACE2-NN-Ig (E), 3 μg/mL NTD antibody (Ab) (F), or plasma sample 9 (at a 1:200 dilution) recognizing only S2 (G). (H) Experiment similar to the one for panel B except that staining was conducted in cells permeabilized with 0.1% saponin. (I and J) S protein expression levels were assessed in cells lysed with dodecyl maltopyranoside and blotted with rabbit anti-S antibody (I) or the same NTD antibody used for panel F (J). The average mean fluorescence intensity values ± SEM from three independent experiments are shown. Statistical significance was analyzed by one-way ANOVA using Dunnett’s multiple-comparison test (B to D) or two-way ANOVA using Sidak’s multiple-comparison test (E to G) (*, *P* < 0.05; **, *P* < 0.005; ***, *P* < 0.0005; ****, *P* < 0.0001; ns, not significant).

Whereas most viruses that are commonly used as a backbone for pseudotyping systems, including human immunodeficiency virus type 1 (HIV-1), murine leukemia virus (MLV), and vesicular stomatitis virus (VSV), bud from the plasma membrane, SARS-CoV-2 buds from the membranes of the ER or the ER-Golgi intermediate compartment (ERGIC) (35). The S protein is therefore designed to be expressed in the intracellular compartments, but when its expression level is high, it is also trafficked to the plasma membrane. Because PV production could benefit from increased S protein expressed on the plasma membrane, we investigated whether CT truncation increased S protein expression on the cell surface. We expressed S-FL and S-dCTs on HEK293T cells via transfection and detected them using convalescent-phase plasma samples derived from coronavirus disease 2019 (COVID-19) patients. Note that whereas all COVID-19 convalescent-phase plasma samples efficiently recognize the S2 subunit, they rarely recognize the S1 subunit (Fig. 2A), likely because the original SARS-CoV-2 did not have much S1 remaining on the virion, owing to its shedding (9). When plasma sample 2, which recognizes both S1 and S2, was used for detection, all S-dCTs, except S-dCT5, were expressed at modestly higher levels than S-FL; albeit modest, the difference was statistically significant (Fig. 2B). However, when plasma sample 9, which recognizes only S2, was used, S-FL and all S-dCTs, except S-dCT5, were expressed at comparable levels (Fig. 2C). Because these data suggest that different S1 levels of S-FL and S-dCTs are responsible for the disparate detection profiles of plasma samples 2 and 9, we also detected S1 alone. To detect only S1, we used hACE2-NN-Ig, the human ACE2 ectodomain that contains enzyme activity-null mutations (H374N and H378N) and is fused to the Fc region of human IgG1 (hIgG1) (21). Figure 2D shows that the S1 levels of S-dCTs, except S-dCT5, detected by hACE2-NN-Ig were approximately 4-fold higher than that of S-FL. These data show that the differences between S-FL and S-dCTs in cell surface expression levels result from their differences in S1, but not S2, contents and that S-dCTs have much higher S1 contents in the spike trimers than S-FL. These data also provide an explanation for the conflicting observations made in previous reports on the cell surface expression of CT-truncated glycoproteins (20, 22–24). Our results demonstrate that depending on which component of the glycoprotein is measured, the expression levels of the CT-truncated glycoproteins could appear to be increased or not increased.

The higher S1 levels observed with S-dCTs than with S-FL prompted us to hypothesize that CT truncation may strengthen the S1-S2 association and, consequently, decrease S1 dissociation from S2. However, because higher hACE2-NN-Ig binding to S-dCTs can result from either a decrease in S1 shedding or an increase in the RBD-up conformation, the conformation that binds the receptor (36–38), we attempted to distinguish these two possibilities by comparing the binding of hACE2-NN-Ig to that of an antibody recognizing the N-terminal domain (NTD) of S1. As this antibody binds the NTD, its binding is unlikely to be affected by the change in the RBD conformation. HEK293T cells were transfected with increasing amounts of an S-dCT19 or S-FL plasmid, their cell surface levels of S1 were measured using hACE2-NN-Ig or the NTD antibody, and their S2 levels were measured using plasma sample 9. Both the hACE2-NN-Ig and NTD antibodies detected severalfold-higher levels of S1 in S-dCT19 than in S-FL at all expression levels (Fig. 2E and F), while the S2 levels of S-dCT19 and S-FL were similar when measured using plasma sample 9 (Fig. 2G). The very similar detection profiles with hACE2-NN-Ig and the NTD antibody of S-FL and S-dCT19 suggest that the RBD-up or -down conformation does not significantly contribute to the differences in the S1 levels but that reduced S1 shedding is likely the major source of the observed S1 differences between S-FL and S-dCTs induced by CT truncation.

In contrast to the other S-dCTs, a low level of S-dCT5 was detected on the cell surface when measured using convalescent-phase plasma sample 2 (Fig. 2B). We thus assessed S-dCT5 expression inside the cell. As the relative level of S-dCT5 compared to other S proteins in permeabilized cells was similar to that on the cell surface (Fig. 2H), we further assessed the S-dCT5 level in cell lysates by Western blotting (WB). Because plasma sample 2 does not efficiently detect S protein in cell lysates, while it does in PVs, a polyclonal anti-S antibody was used for blotting. As shown in Fig. 2I, while comparable levels of the uncleaved S band were observed for all S proteins, indicating that S-dCT5 is expressed comparably to the others, much lower levels of the S1 and S2 bands were observed for S-dCT5, suggesting that they may be rapidly degraded once cleaved. Because this antibody detects S1 only very weakly, we used the NTD antibody to better visualize the S1 bands. Figure 2J shows that the S1 bands in all S-dCTs, except S-dCT5, are stronger than that in S-FL. These data are consistent with our conclusion drawn from cell surface expression and confirm that S-dCTs, except S-dCT5, retain higher levels of S1 than S-FL owing to reduced S1 shedding.

### Cytoplasmic tail truncation dramatically enhances functional S protein incorporation into PV particles.

**FIG 3:**
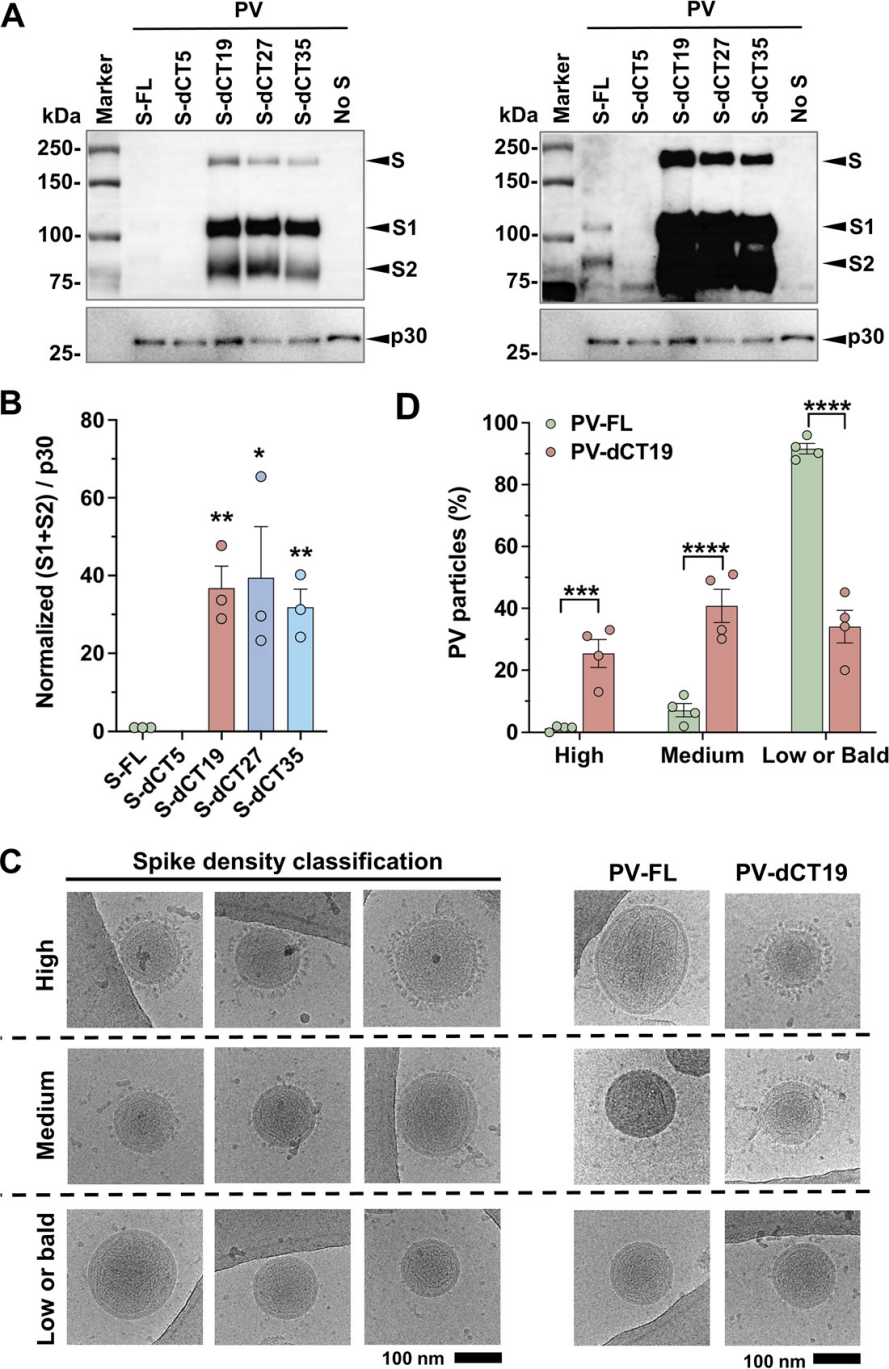
Cytoplasmic tail truncation dramatically enhances S protein incorporation into PV particles. (A) PVs bearing the indicated S variants and produced from HEK293T cell transfection were pelleted through a layer of 30% of sucrose and analyzed by Western blotting. The S, S1, and S2 bands were detected using convalescent-phase plasma sample 2 at a 1:200 dilution. (Left) Blot representative of results from three independent experiments. The S1 and S2 bands of S-FL and S-dCT5 are too weak to be detected. (Right) A much longer exposure of the same blot shown on the left. The S1 and S2 bands of S-FL, but not those of S-dCT5, are visible. (B) Sum of the S1 and S2 band intensities of the Western blot shown in panel A normalized to the intensity of the p30 band in the same blot. Shown are the averages from three independent experiments. (C, left) As both PV-FL and PV-dCT19 exhibit heterogeneous spike densities, to provide an objective guideline for semiquantitative evaluation, three representative cryo-EM images were selected for each of the high-, medium-, and low/bald-spike-density groups. (Right) Images selected separately from PV-FL and PV-dCT19 for each category. Note that there are only one high-density and a few medium-density particles among 61 PV-FL particles. (D) A total of 149 PV particles (61 PV-FL and 88 PV-dCT19 [shown in Fig. S1 in the supplemental material]) were evaluated by four individuals and categorized into the high-, medium-, and low/bald-density groups using the criteria described above for panel C. Mean values ± SEM are shown. Statistical significance was analyzed by an unpaired *t* test (B) and two-way ANOVA using Sidak’s multiple-comparison test (D) (*, *P* < 0.05; **, *P* < 0.005; ***, *P* < 0.005; ****, *P* < 0.0001).

As the S protein mediates receptor attachment, the quantity and quality of spike trimers on the virion determine virus and PV infectivity. Thus, we measured the S protein density on PV-FL and PV-dCTs, which were pelleted through a sucrose layer (“sucrose-pelleted PV”), by WB analyses, using plasma sample 2, which recognizes both S1 and S2 (Fig. 2A). When the same numbers of PV particles were analyzed, as supported by the comparable amounts of p30, the MLV Gag protein, the intensities of the S1 and S2 bands of all PV-dCTs, except PV-dCT5, were dramatically increased compared to those of PV-FL (Fig. 3A, left), which were barely detectable only with a much longer exposure of the same blot (Fig. 3A, right). These data demonstrate that CT truncation leads to the much more efficient incorporation of the S protein into PV particles.

To confirm that the increased intensities of the S1 and S2 bands of PV-dCTs assessed by WB analyses (Fig. 3A and B) actually reflect an increased spike density on the virion, we visualized PV-FL and PV-dCT19 by cryo-electron microscopy (cryo-EM). We focused on PV-dCT19 because it exhibits the highest infectivity and virion spike density. Examined by cryo-EM, both PV-FL and PV-dCT19 were quite heterogeneous with respect to their spike densities. Although PV-dCT19 particles generally exhibited higher spike densities than PV-FL particles, to analyze them in a semiquantitative way, we sorted PV-FL and PV-dCT19 cryo-EM particles into high-, medium-, and low (or bald)-spike-coverage groups (Fig. 3C). Three cryo-EM images, each representing the three categories, are shown in Fig. 3C, left. Using these criteria, cryo-EM images of 61 PV-FL and 88 PV-dCT19 particles (see Fig. S1 in the supplemental material) were sorted with four independent counts. Note that we found only one high-density and two medium-density PV-FL particles. Most PV-FL particles belonged to the low-surface-density group. Specifically, 1.3% and 7.1% of the PV-FL particles belonged to the high- and medium-density groups, respectively, while the majority, 91.6%, had low or undetectable levels of spike (Fig. 3D). In contrast, PV-dCT19 virions were more evenly distributed among the high-, medium-, and low-surface-density groups (Fig. 3D): high at 25.4%, medium at 40.6%, and low/bald at 34.0%. These cryo-EM data are consistent with the WB results and clearly demonstrate that PV-FL overall has a much lower virion spike density than does PV-dCT19.

### Cytoplasmic tail truncation significantly enhances functional S protein on PV particles.

**FIG 4:**
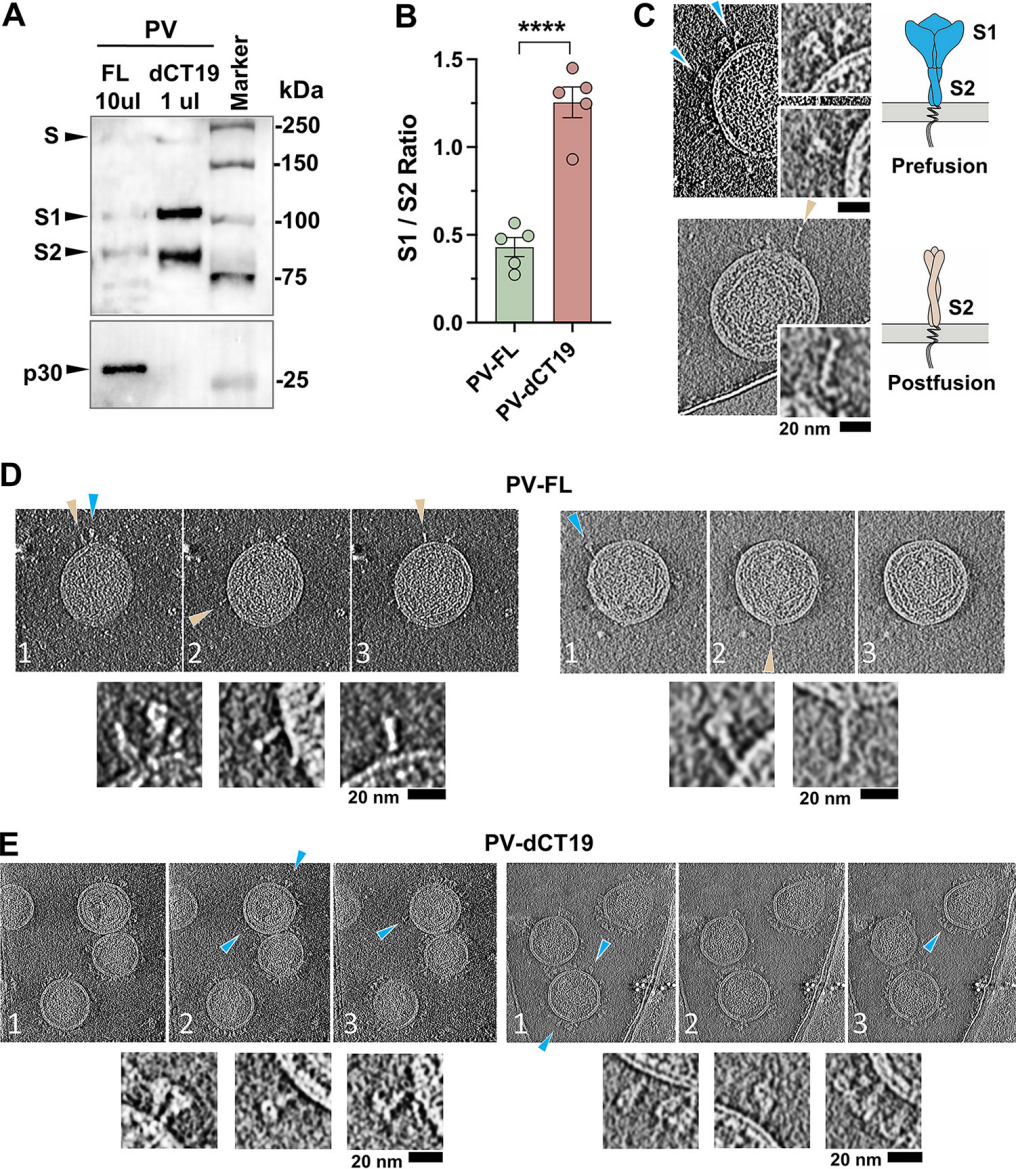
Cytoplasmic tail truncation significantly enhances functional S protein on PV particles. (A) The same PVs shown in Fig. 3A but with PV-FL compared to 10-times-less PV-dCT19 to more accurately measure their S1/S2 ratios. (B) Mean S1/S2 ratios ± SEM of PV-FL and PV-dCT19 analyzed from five Western blots performed with three sets of independently prepared PV batches. Statistical significance was analyzed by an unpaired *t* test (****, *P* < 0.0001). (C) Prefusion (top) (blue arrows) and postfusion (bottom) (light-brown arrow) conformations of spike trimers on sucrose-pelleted PVs examined by cryo-EM tomography. Schematic representations of these two spike conformations are presented at the right. (D) Sequential slices, indicated by the numbers at the bottom left, of PV-FL cryo-electron tomograms, with a mix of prefusion (blue arrows) and postfusion (light-brown arrows) S conformations, enlarged at the bottom. (E) Sequential slices, indicated by the numbers at the bottom left, of PV-dCT19 cryo-electron tomograms, with mostly the prefusion (blue arrows) S conformation, enlarged at the bottom. See also Fig. S2 in the supplemental material.

Much higher S1 levels were detected on PV-dCTs (Fig. 3A and B) as well as S-dCTs on the cell surface. To confirm that the higher level of S1 indeed reflects the more efficient retention of S1 on PV-dCTs, we analyzed the S1/S2 ratio of PV-dCTs and PV-FL. Because of the much lower S density of PV-FL, a large amount of PV-FL was compared to a much smaller amount of PV-dCTs in order to detect both the S1 and S2 bands in both PVs. As Fig. 4A and its quantification in Fig. 4B show, the S1/S2 ratio of PV-dCT19 is approximately 2.5-fold higher than that of PV-FL. This result is consistent with the differences that we observed with cell surface-expressed S-FL and S-dCTs (Fig. 2D, E, and F) and again indicates that CT truncation contributes to the greater retention of S1.

To understand the three-dimensional organization of the S protein on the surface of PVs, we performed cryo-EM tomography with PV-FL and PV-dCT19 (Fig. 4 and Fig. S2). On some of the PV surfaces, we were able to distinguish the prefusion trimers, which look like half-blossomed flowers, from the postfusion trimers, which look like thin sticks, as a result of S1 shedding and the conversion of S2 to a helical-bundle conformation (Fig. 4C) (37–39). Sequentially slabbing through the three-dimensional tomogram slices, we observed that most PV-FL particles have extremely low coverage of surface proteins and that the majority of the spike trimers are in the postfusion conformation, with a subset being in the prefusion conformation (Fig. 4D). In contrast, on PV-dCT19, prefusion spikes can be abundantly identified amid closely spaced clusters of proteins on the PV surface resolved in three dimensions (Fig. 4E). While the high density of protein on the PV-dCT19 particles could mask the presence of some postfusion spikes, and only a portion of the spike conformations are clearly identifiable, many spike trimers appear to be in the prefusion conformation. The higher abundance of prefusion spike trimers on PV-dCT19 is consistent with the presence of intact, functional S-dCTs on the cell surface (Fig. 2) and the high S1/S2 ratios observed in WB analyses of PV-dCTs relative to PV-FL (Fig. 4A). Taken together, these data demonstrate that CT truncation enhances S protein incorporation into PVs and S1 retention in the spike, leading to much higher numbers of prefusion spikes on the pseudovirion, thus providing an explanation for the dramatic increase in PV-dCT infectivity.

## DISCUSSION

CT truncation of virus entry glycoproteins has been widely used to enhance the infectivity of various PVs. Although several potential explanations were offered, the underlying mechanism for the greatly enhanced PV infectivity is still unclear. Increased cell surface expression of viral glycoproteins upon CT truncation was proposed as an explanation for the enhanced PV infectivity, but conflicting results were also reported (20, 22–24). Furthermore, even if cell surface expression is increased, the degree of the increase is modest and thus would not be sufficient to explain the dramatic change in PV infectivity. Increased fusogenicity of the glycoproteins induced by CT truncation was also proposed for measles, murine leukemia, vesicular stomatitis, and Nipah viruses (18, 20, 23, 25–27, 33), and a conformational change in the ectodomain induced by inside-out signaling was proposed to explain such increased fusogenicity (20, 25, 27).

**FIG 5:**
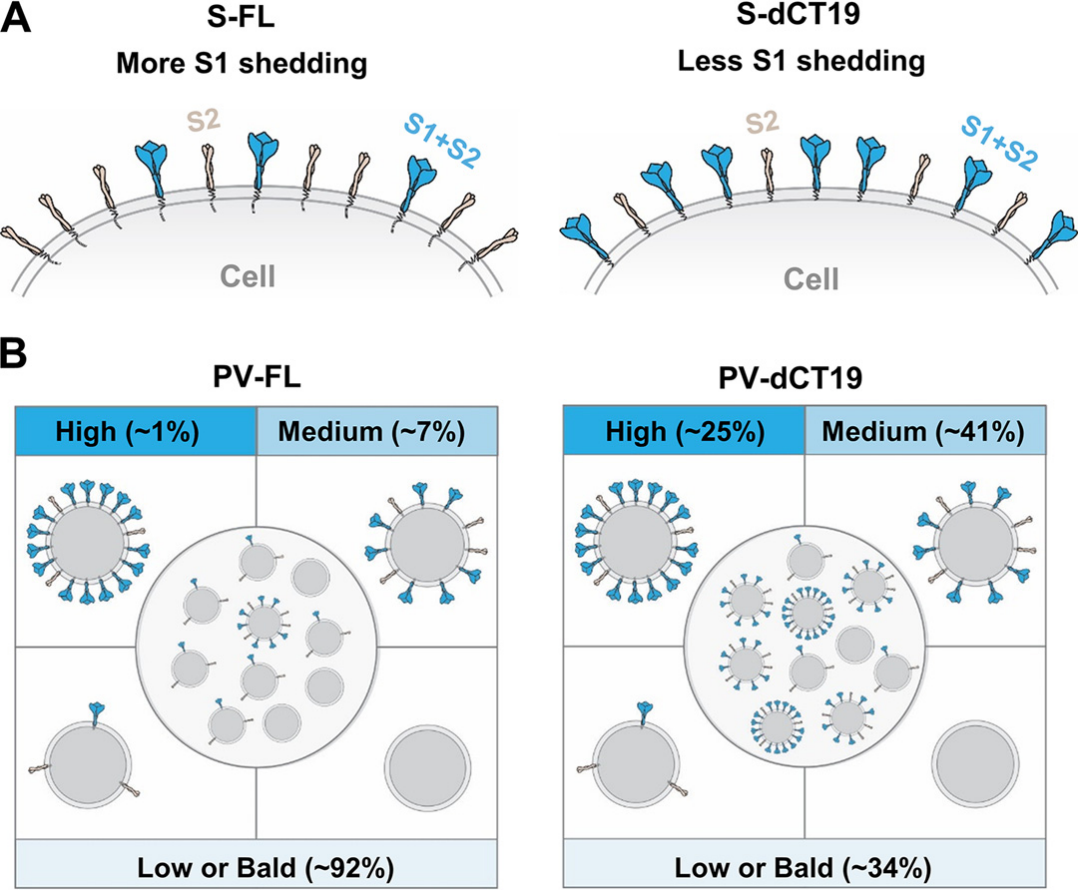
Schematic representation of increased functional spikes on the cell surface and in pseudoviruses upon CT truncation of the S protein. (A) Higher levels of functional S-dCTs are observed on the cell surface, while S2 levels are similar between S-dCTs and S-FL. Functional spikes (in blue), assessed using ACE2-NN-Ig or NTD antibody (Fig. 2D to F), contain both S1 and S2 in the prefusion conformation. Nonfunctional spikes (in light brown), measured using plasma sample 9, which recognizes only S2 (Fig. 2C and G), consist of only S2 in the postfusion conformation. (B) PV-dCTs exhibit much higher levels of functional spikes than PV-FL. The diagrams of four PVs represent those with a high, medium, or low spike density or bald virions. The group of 10 PV particles in the center for PV-FL and PV-dCTs reflects the approximate composition of each PV population with respect to the virion spike density. The proportion of each spike density group in the PV-FL and PV-dCT19 populations was assessed using cryo-EM micrographs (Fig. 3C and D; see also Fig. S1 in the supplemental material).

Our study here shows that the cell surface expression of CT-truncated SARS-CoV-2 S protein could lead to different outcomes depending on which subunit, S1, S2, or both, is measured (Fig. 5A). If S2 is measured, no significant difference is detected, while a modest increase is observed if both S1 and S2 are measured. However, if only S1 is measured, a much higher S1 level is observed with S-dCT19, -27, and -35 than with S-FL. These data provide an explanation for the conflicting reports on the cell surface expression of the CT-truncated viral entry glycoproteins (20, 22–24).

Our study further identifies two different mechanisms underlying the dramatically increased infectivity of SARS-CoV-2 PVs carrying a CT truncation. Through biochemical and cryo-electron microscopy, we show that CT truncation promotes S protein incorporation into PVs and enhances S1 retention on the S protein. These two events together result in much higher numbers of functional spikes in PV-dCT19, -27, and -35 (Fig. 5B), leading to a dramatic increase in their infectivity. Our results on the increased S1 retention on S-dCTs are consistent with previous reports in which increased fusogenicity upon CT truncation was observed for the entry glycoproteins of various viruses (18, 25–27). Those studies were conducted using syncytium formation assays, and thus, a higher level of the surface subunit of a glycoprotein expressed on the cell surface would have resulted in more efficient syncytium formation. Although further studies are necessary, the increased S1 retention on S-dCTs may arise from the enhanced flexibility of the S2 subunit, which accommodates a more stable S1-S2 interaction.

Our data also show that the differences in spike density induced by CT truncation are much greater in PVs than on the cell surface; while the S2 levels are similar between S-FL and S-dCTs on the cell surface (Fig. 2C), they are much higher in PV-dCTs than in PV-FL (Fig. 3A). A higher spike density in PV-dCTs would be possible if S-dCTs are more enriched in lipid rafts than S-FL because many viruses bud from this microdomain. However, S-dCT27 and S-dCT35 lack some or all of the motifs for palmitoylation, a modification that promotes protein trafficking into lipid rafts (40), and thus, it is unlikely that S-dCTs are enriched in lipid rafts and more efficiently incorporated into the virion. A more likely explanation is that S-dCTs can be more freely incorporated into PVs because they lack the binding motif for Ezrin-Moesin-Radixin proteins that anchor membrane proteins to the cellular cytoskeleton (41). An alternative explanation is that the steric hindrance formed between the structural proteins of PVs (Gag or matrix protein) and the large CT of S-FL makes it difficult to be incorporated into PVs, whereas the smaller CT of S-dCTs allows more efficient incorporation into PVs, as we previously proposed (21).

## MATERIALS AND METHODS

### Plasmids.

The SARS-CoV-2 S protein gene was codon optimized and synthesized by Integrated DNA Technologies based on the protein sequence (Wuhan-Hu-1 strain [GenBank accession number YP_009724390]) and cloned into the pCAGGS vector (42). The genes for cytoplasmic-tail-truncated variants of the SARS-CoV-2 S protein were also synthesized and cloned into the pCAGGS vector. None of these genes contain a tag. The retroviral vector pQCXIX (Clontech), encoding enhanced green fluorescent protein (eGFP) or firefly luciferase (FLuc), and the plasmid expressing the MLV Gag and Pol proteins or the G protein of VSV (VSV-G) were previously described (43). An hTMPRSS2 expressor plasmid was constructed by cloning its residues 1-492 (GenBank accession number NP_005647) into the retroviral vector pQCXIB (44). The hACE2 expressor plasmid was constructed by cloning its residues 20 to 805 (GenBank accession number NM_021804) downstream of the mouse ACE2 signal sequence (MSSSSWLLLSLVAVTTAQS) and the Myc tag sequence into the retroviral vector pQCXIP. The expression plasmid for hACE2-NN-Ig was previously described (45): the hACE2 ectodomain fragment (residues 20 to 615 [GenBank accession number NM_021804]) containing the H374N and H378N mutations was cloned into pcDNA3.1 containing the CD5 signal sequence and the Fc region of human IgG1.

### Cells.

Human embryonic kidney HEK293T cells were obtained from the ATCC and maintained in high-glucose Dulbecco’s modified Eagle’s medium (DMEM) supplemented with 10% fetal bovine serum (FBS) at 37°C with 5% CO_2_. HEK293T cells transduced to stably express hACE2 and hTMPRSS2 (hACE2/hTMPRSS2-293T cells) or mock transduced (Mock-293T cells) were selected and maintained in medium supplemented with 1 μg/mL puromycin and 10 μg/mL blasticidin (InvivoGen). The transduction vectors for hACE2 and hTMPRSS2 were produced by transfecting pQCXIP-hACE2 or pQCXIB-hTMPRSS2 into HEK293T cells together with the plasmid encoding murine leukemia virus Gag-Pol and the plasmid encoding the G protein of vesicular stomatitis virus. The vector for mock transduction was produced similarly using the empty pQCXIP or pQCXIB plasmid.

### MLV PV production and sucrose pelleting.

HEK293T cells at ~60% confluence in T75 flasks were transfected using a calcium-phosphate method with 24 μg of total DNA at a ratio of 5:5:1 (by mass) of the retroviral vector pQCXIX encoding eGFP or FLuc, the plasmid expressing MLV Gag and Pol proteins, and the plasmid expressing either the full-length S protein or the truncated version of the S protein of SARS-CoV-2 (Wuhan-Hu-1). Transfected cells were washed at 6 h posttransfection and replenished with 10 mL DMEM supplemented with 10% FBS. The PV-containing culture supernatants were collected at 43 h posttransfection, cleared through 0.45-μm filters, and either immediately aliquoted and stored at −80°C or used for entry experiments.

For sucrose-pelleted PV preparation, 10 mL of the cleared culture supernatants containing PVs precleared by centrifugation in a TX-400 swinging-bucket rotor at 3,000 rpm for 10 min followed by filtration through 0.22-μm filters was loaded onto 2 mL of 30% sucrose (catalog number S7903; Sigma-Aldrich) in NTE buffer (120 mM NaCl, 20 mM Tris, 2 mM EDTA [pH 8.0]) and centrifuged at 50,000 × *g* in an SW41 rotor for 2 h at 10°C. The PV pellets were resuspended in 20 μL NTE buffer with gentle shaking on ice and either used immediately or aliquoted and frozen at −80°C.

### PV quantification.

PVs were quantified by RT-qPCR using primers and a probe that target the cytomegalovirus (CMV) promoter. Culture supernatants containing PVs were treated with 100 μg/mL RNase A for 1 h at 37°C to degrade RNAs that were not packaged inside the virion, and RNA was extracted with TRIzol and GlycoBlue coprecipitant and digested for 30 min at 37°C with DNase I at 1 IU per 1 μg extracted RNA. DNase I was inactivated by incubation for 10 min at 65°C with EDTA added to a final concentration of 5 mM. DNase-treated RNA was reverse transcribed using a high-capacity cDNA reverse transcription kit (catalog number 4374966; Applied Biosystems). qPCR was performed, using Luna universal probe qPCR master mix (catalog number M3004E; New England BioLabs) with a known quantity of the pQCXIX vector (Clontech), to generate standard curves, and data were collected with CFX Manager 3.1 (Bio-Rad). The primers and probe were synthesized by Integrated DNA Technologies (sense primer 5′-TCACGGGGATTTCCAAGTCTC-3′, antisense primer 5′-AATGGGGCGGAGTTGTTACGAC-3′, and probe 5′-FAM [6-carboxyfluorescein]-AAACAAACT-[ZEN]-CCCATTGACGTCA-IBFQ-3′).

### PV entry assay in hACE2-hTMPRSS2-293T cells.

A PV entry (transduction) assay was performed by incubating Mock-293T or hACE2/hTMPRSS2-293T cells on 48-well plates with PVs (5 × 10^8^ genome copies in 200 μL per well) for 1 h at 37°C in a CO_2_ incubator. Medium was replaced with DMEM containing 10% FBS. The entry levels of PVs expressing firefly luciferase were assessed by measuring luciferase activity using the Luc-Pair firefly luciferase HS assay kit (catalog number LF009; GeneCopoeia) and reading the plates on the SpectraMax paradigm multimode detection platform using SoftMax Pro 6.3 (Molecular Devices).

### Screening of human plasma.

Deidentified plasma samples were obtained by the Allergy, Asthma, and Immunology Specialists of South Florida, LLC, in mid-2020 for COVID-19 serotyping, and these were exempt (IRB-20-7580) from human subject research under CFR 45.101(b). These plasma samples were screened by Western blotting at a 1:200 dilution for their ability to recognize the S1 and S2 subunits of the SARS-CoV-2 S protein on sucrose-pelleted PV-dCT19. The blot was visualized using 1:10,000-diluted goat anti-human IgG conjugated with horseradish peroxidase (HRP) (catalog number 109-035-098; Jackson ImmunoResearch).

### Cell surface and total expression of the spike protein.

HEK293T cells at approximately 70% confluence in 6-well plates were transfected with 0.3 μg, unless indicated otherwise, of the plasmid expressing the indicated S protein variant. Cells were detached at 42 h posttransfection in phosphate-buffered saline (PBS) containing 2 mM EDTA. Approximately 1 × 10^6^ cells were fixed with a 2% formaldehyde solution in PBS for 30 min on ice and blocked with PBS containing 2% bovine serum albumin (BSA) (Sigma-Aldrich) and 5% goat serum (Gibco) for 30 min on ice. These cells were then incubated on ice for 90 min in 100 μL of PBS containing either SARS-CoV-2 convalescent-phase plasma at a 1:200 dilution, 3 μg/mL of purified hACE2-NN-Ig (9), or 3 μg/mL of anti-NTD monoclonal antibodies (catalog number SPD-M121; Acro Biosystems), followed by 1:600-diluted anti-hIgG–Alexa Fluor 647 (AF647) (catalog number 109-605-003; Jackson ImmunoResearch). Stained cells were analyzed using an Accuri flow cytometer (BD Biosciences) equipped with the HyperCyt autosampler (IntelliCyt) and ForeCyt 6.2R3 software (IntelliCyt). To measure the total expression levels of the S protein, aliquots of the same cells were permeabilized with 0.1% saponin (Alfa Aesar) in PBS for 10 min at room temperature and subjected to staining on ice as described above.

### Western blot analysis of S protein in cell lysates and on pseudovirions.

For the detection of S protein bands in the cell lysates, HEK293T cells in 6-well plates were transfected to express S-FL or S-dCT5 and lysed with 0.2 mL of PBS containing 0.5% dodecyl maltopyranoside (Anatrace) and a protease inhibitor cocktail (catalog number A32955; Thermo Scientific). Fifteen microliters of the lysates was analyzed by WB using rabbit anti-S protein antibody (catalog number NR-52947; BEI Resources) at a 1:5,000 dilution or 1 μg/mL of NTD antibody (catalog number SPD-M121; Acro Biosystems) For the analyses of the S protein density on the virion, sucrose-pelleted PVs were analyzed by SDS-PAGE and WB. Unless indicated otherwise, 5 μL PVs was analyzed in each WB analysis. PV proteins were separated on a 4 to 12% Bis-Tris gel (catalog number NW041122; Life Technologies) and transferred to a polyvinylidene difluoride (PVDF) membrane. The membrane was blocked with 5% milk in 1× Tris-buffered saline containing 0.1% Tween 20 for 1 h and blotted with human SARS-CoV-2 convalescent-phase plasma at a 1:200 dilution to detect the S protein bands or 1 μg/mL anti-p30 MLV Gag antibody (catalog number ab130757; Abcam) to detect p30 bands as a PV quantity control. S protein bands were detected using 10 ng/mL mouse anti-human IgG antibody conjugated with polymerized HRP (catalog number 61R-I166AHRP40; Fitzgerald), and p30 bands were detected using 1:10,000-diluted goat anti-mouse IgG–HRP polyclonal antibody (catalog number 115-036-062; Jackson Immuno Laboratory). Bands were visualized using the SuperSignal West Atto ultimate-sensitivity substrate (catalog number A38555; Thermo Scientific), and the band intensities were measured using Image Lab software (Bio-Rad).

### Cryo-EM of full-length and dCT19 spike proteins.

For cryo-EM samples, 300-mesh R2/2 copper Quantifoil grids (Electron Microscopy Sciences [EMS]) were negatively glow discharged for 30 s. The PV sample (3.5 μL) was applied onto the grid and incubated for 30 s, followed by plunge-freezing into liquid ethane with a Vitrobot Mark IV instrument (100% humidity, 4°C, and 4.5 s per blot). The grids were imaged on an FEI Tecnai T12 120-kV electron microscope.

For cryo-electron tomography, grids were prepared as described above, with the addition of 10-nm gold beads at a ratio of 14:1 (vol/vol) before plunge-freezing. Dose-symmetric tilt series (to ±48° with 3° increments) were collected on a Titan Krios instrument with a K3 detector (total dose of 100 e^−^/Å^2^; 7 frames per angle) and a 20-eV energy filter. The data set was motion corrected with MotionCor2 (46), reconstructed with IMOD (47), denoised with Topaz (48), and processed using ImageJ software.

### Statistical analysis.

All of the statistical details of specific experiments, which included the statistical tests used, numbers of samples, mean values, standard errors of the means (SEM), and *P* values derived from the indicated tests, are described in the figure legends and shown in the figures. Statistical analyses were conducted utilizing GraphPad Prism version 8.0 (GraphPad Software Inc.). Triplicate and other replicative data are presented as means ± SEM. A *P* value of <0.05 was considered to be statistically significant. For comparisons between two treatments, Student’s *t* test (unpaired) was used. For comparisons of each group with the mean of every other group within a data set containing more than two groups, either one-way analysis of variance (ANOVA) with Dunnett’s multiple-comparison test or two-way ANOVA with Sidak’s multiple-comparison test was used.
