# Supplementary material for: Cytoplasmic Tail Truncation Stabilizes S1-S2 Association and Enhances S Protein Incorporation into SARS-CoV-2 Pseudovirions
Source: J Virol. 2023 Feb 15;97(3):e01650-22. doi: 10.1128/jvi.01650-22 (PMC10062125; doi:10.1128/jvi.01650-22)
Supplement: Supplemental file 1 — Fig. S1 and S2. Download jvi.01650-22-s0001.pdf, PDF file, 0.3 MB [file jvi.01650-22-s0001.pdf]

SUPPLEMENTARY MATERIAL

All cryo-EM images used for categorizing PVs into different spike-density groups in Fig. 3C and 3D are shown in Fig. S1. Extra tomograms of prefusion and postfusion spikes on PV-FL and PV-dCTs presented in Fig. 4C-4E are shown in Fig. S2.

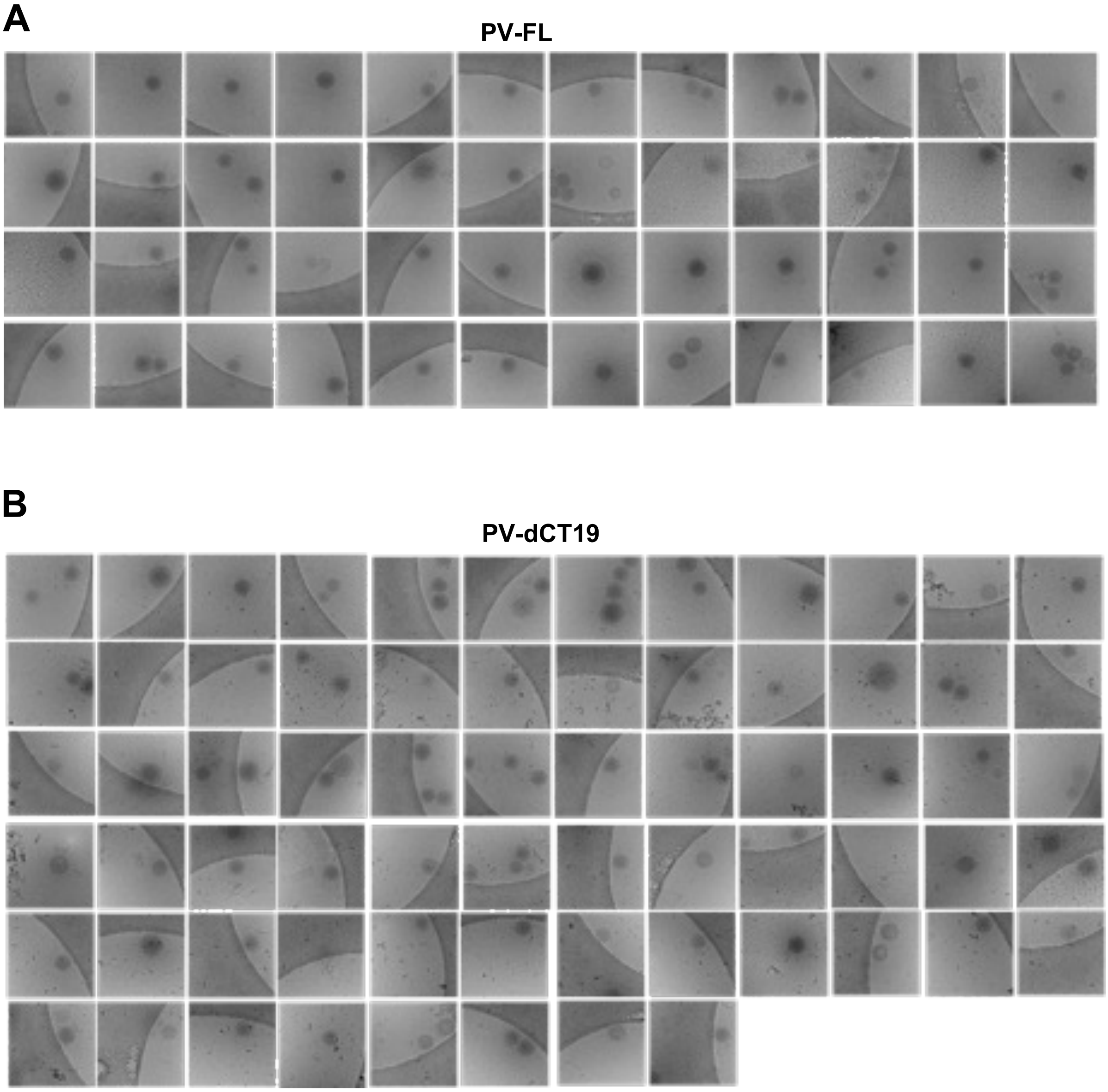

FIG S1 (related to FIG 3). Pseudovirion spike density is heterogenous but much higher in PV-dCT19 than PV-FL. Cryo-EM images of all of 61 PV-FL and 88 PV-dCT19 particles analyzed in Fig. 3D are shown.

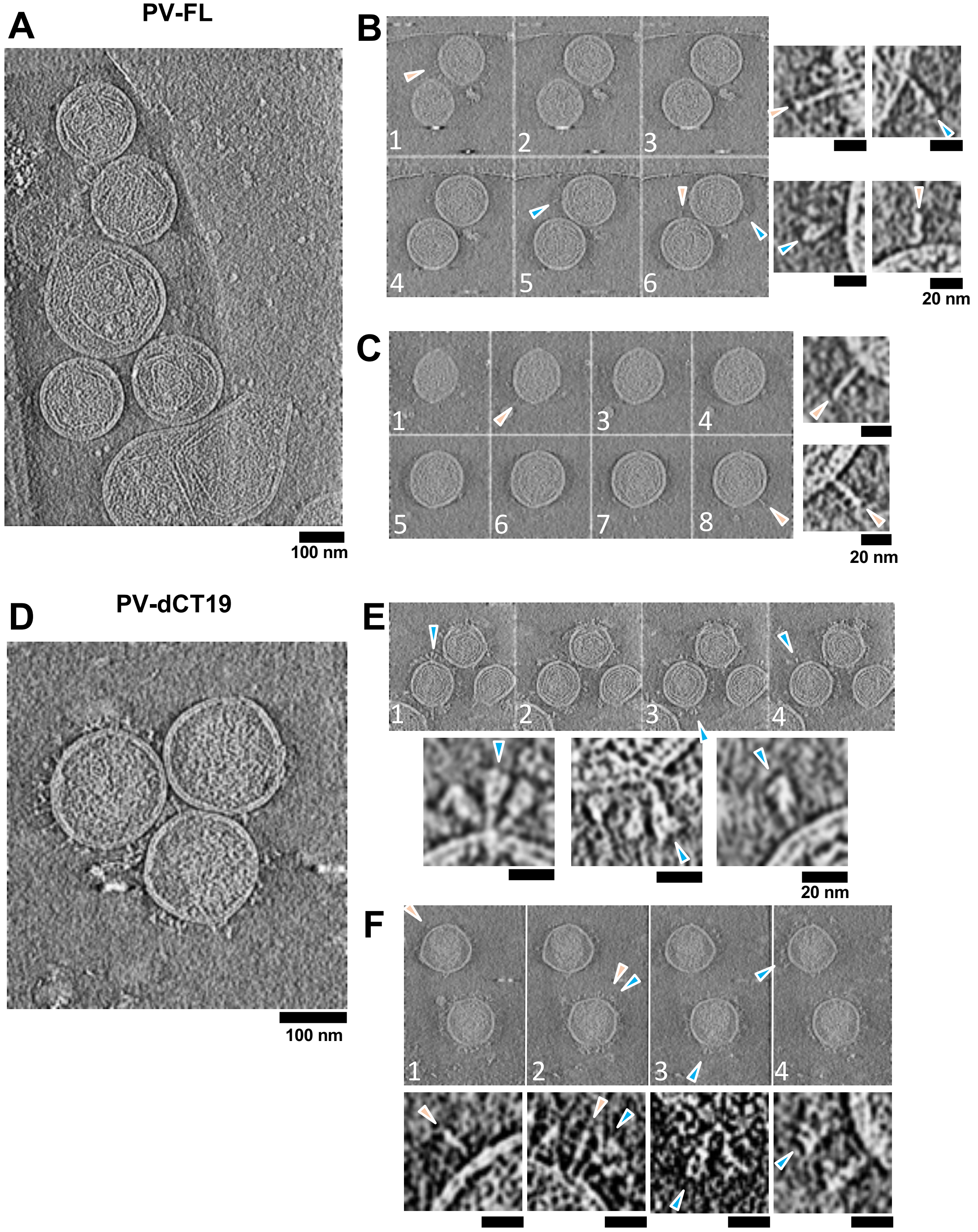
A
PV-FL
100 nm
B
1
2
3
4
5
6
20 nm
C
1
3
4
5
6
7
8
20 nm
D
PV-dCT19
100 nm
E
1
2
3
4
20 nm
F
1
2
3
4

12

13

FIG S2 (related to FIG 4). Additional reconstructed tomograms of PV-FL and PV-dCT19. Tomograms of PV-FL (A-C) and PV-dCT19 (D-F). (A, D) Tomogram slices on the left show typical field of view. (B,C) Slices through tomograms for PV-FL. (E,F) Slices through tomograms for PV-dCT19. The pre-fusion (blue arrows) and post-fusion (light brown arrows) conformational states of S protein are indicated and enlarged. Higher magnification images on the right can be identified by the arrowhead of the same color and direction as that in the original image. Scale bars correspond to dimensions as indicated.
